# Microtubule detyrosination links inflammasome activation to apoptotic cell death in macrophages upon influenza A virus infection

**DOI:** 10.1128/jvi.01847-25

**Published:** 2025-12-09

**Authors:** Joyeeta Kar, Mikako Hirohama, Kotono Nakayama, SangJoon Lee, Atsushi Kawaguchi

**Affiliations:** 1 Graduate School of Comprehensive Human Sciences, University of Tsukuba 90521, Tsukuba, Japan; 2 Department of Infection Biology, Institute of Medicine, University of Tsukuba 38515, Tsukuba, Japan; 3 School of Medicine, University of Tsukuba 38515, Tsukuba, Japan; 4 Department of Biological Sciences, Ulsan National Institute of Science and Technology (UNIST) 131639, Ulsan, Republic of Korea; 5 Transborder Medical Research Center, University of Tsukuba 622602, Tsukuba, Japan; 6 Microbiology Research Center for Sustainability, University of Tsukuba 623473, Tsukuba, Japan; 7 Center for Quantum and Information Life Sciences, University of Tsukuba 13121, Tsukuba, Japan; University of North Carolina at Chapel Hill, Chapel Hill, North Carolina, USA

**Keywords:** detyrosination, apoptosis, microtubule, inflammasome, influenza virus

## Abstract

The tubulin code, defined by diverse post-translational modifications of microtubules, fine-tunes microtubule dynamics, regulating downstream cellular signaling. Among these, detyrosination of α-tubulin has long been recognized, yet its upstream regulation and physiological roles in viral infection remain unclear. Here, we show that influenza A virus (IAV) infection induces heterogeneous cell death morphologies in macrophages, including pyroptotic “ghost” cells and apoptotic beaded apoptopodia. Beaded apoptopodia were enriched with detyrosinated α-tubulin, a long-lived, stable microtubule modification generated by enzymatic removal of the C-terminal tyrosine residue. We found that the detyrosination was dependent on caspase-1-mediated inflammasome signaling. Pharmacological inhibition of vasohibin-1 (VASH1) suppressed detyrosination without affecting viral replication, identifying VASH1, but not VASH2, as the predominant carboxypeptidase responsible for this modification upon IAV infection. Overexpression of VASH1 enhanced detyrosination and shifted cell death from pyroptosis toward apoptosis. These findings uncover a cytoskeletal pathway that modulates inflammasome signaling toward immunologically silent apoptosis, thereby limiting inflammatory cell lysis. This mechanism highlights the physiological significance of VASH1-mediated detyrosination in shaping host responses to viral infection.

**IMPORTANCE:**

Programmed cell death is an essential host response to viral infection, but whether infected macrophages undergo inflammatory or non-inflammatory forms of death has important consequences for disease progression. In this study, we found that influenza A virus infection induces a modification of microtubules known as detyrosination, which stabilizes their structure. This change was driven by the host enzyme vasohibin-1 through activation of the inflammasome, a key signaling complex that normally promotes inflammatory cell death. Remarkably, enhanced detyrosination shifted dying cells away from inflammatory membrane rupture toward apoptosis, an immunologically silent cell death pathway that preserves membrane integrity. Our findings identify microtubule detyrosination as a stress-induced host response during influenza A virus infection, highlighting a novel mechanism by which cytoskeletal modification influences the outcome of infection.

## INTRODUCTION

Programmed cell death is a critical component of the host defense response against viral infection (1). Depending on the context, infected cells may undergo various forms of programmed cell death, such as apoptosis and pyroptosis, with distinct molecular mechanisms and immunological outcomes (2, 3). Among these, pyroptosis is regulated by the inflammasome complex, typically consisting of a pattern recognition receptor such as NLRP3, an adaptor protein ASC, and caspase-1 (4). Pyroptosis is a lytic form of cell death characterized by cell swelling and plasma membrane rupture, leading to the secretion of pro-inflammatory cytokines such as interleukin (IL)-1β and IL-18 (5, 6). In contrast, apoptosis is a non-lytic and immunologically silent cell death regulated by caspase-3 (7). Apoptosis forms discrete membrane vesicles, known as apoptotic bodies, through cytoskeletal reorganization (8).

The cytoskeleton, mainly microtubules and actin filaments, serves as both sensors and effectors of programmed cell death (9, 10). F-actin can rearrange into a cortical contractile ring preceding mitochondrial outer-membrane permeabilization and caspase-3 activation, promoting apoptosis (8, 11). In pyroptosis, depolymerization of actin filaments enhances NLRP3 inflammasome activation by relieving its actin-mediated inhibition (12). Microtubules are also implicated in determining cell death pathways; paclitaxel-stabilized microtubules promote apoptosis, while depolymerization with colchicine favors necrotic cell death (13). Furthermore, microtubules mediate the clustering of death receptor 5 into an apoptotic microtubule network that assists membrane blebbing and orderly fragmentation (14). However, the modulators that regulate the cross talk among programmed cell death pathways remain poorly understood (15).

Microtubules are dynamic cytoskeletal filaments whose assembly and functions are fine-tuned by diverse post-translational modifications (PTMs) of their tubulin subunits, called “tubulin code.” Key modifications such as detyrosination/tyrosination cycles, acetylation, polyglutamylation, and others (e.g., glycylation and phosphorylation) modulate microtubule dynamics and interactions with effector proteins (16). For instance, detyrosinated and acetylated microtubules are typically long-lived and stable, whereas tyrosinated microtubules mark dynamic, short-lived filaments (17, 18). These PTMs regulate microtubule mechanics and surface properties, regulating the engagement of motor proteins and microtubule-associated proteins (19, 20). Notably, stable microtubules enriched in detyrosinated or acetylated tubulin are preferentially recognized by specific kinesin motors for cargo transport (21, 22). Among these, the detyrosination of α-tubulin (detyr-tubulin)—a process involving the enzymatic removal of the C-terminal tyrosine residue—has been recognized for decades but remains mechanistically understudied. Detyr-tubulin is induced by vasohibin-1 (VASH1) or vasohibin-2 (VASH2) in complex with the chaperone protein, small vasohibin-binding protein (SVBP) (23–25). In contrast, tubulin-tyrosine ligase (TTL) reverses this process by re-adding the C-terminal tyrosine residue (26, 27). The mechanistic downregulation of TTL by epithelial-to-mesenchymal transition increases the detyr-tubulin and promotes protrusive “micro-tentacles” that facilitate tumor cell survival in the bloodstream (28). Recent evidence suggests that the tubulin code can modulate downstream signaling pathways: for example, elevated tubulin acetylation can enhance cellular resistance to paclitaxel-induced apoptosis (29), and detyrosination inhibition has been shown to impair autolysosome formation (30). However, the connection between the tubulin code and programmed cell death pathways during viral infection remains unclear. Elucidating this mechanism is essential to understanding how the cytoskeleton shapes cell fate decisions in host-virus interactions.

In this study, we investigated the morphological diversity of cell death in influenza A virus (IAV)-infected macrophages and examined how microtubule detyrosination regulates pyroptosis and apoptosis. We found that apoptotic cells showed long, beaded protrusions enriched with detyr-tubulin. This detyrosination was mediated by VASH1 in a caspase-1-dependent manner. VASH1 overexpression increased detyrosination and shifted the mode of programmed cell death from inflammasome-mediated pyroptosis to apoptosis. Our results uncover a novel connection between the tubulin code, inflammasome signaling, and cell fate decision in virus-infected macrophages.

## RESULTS

### IAV infection induces distinct cell death morphologies in macrophages

**Fig 1:**
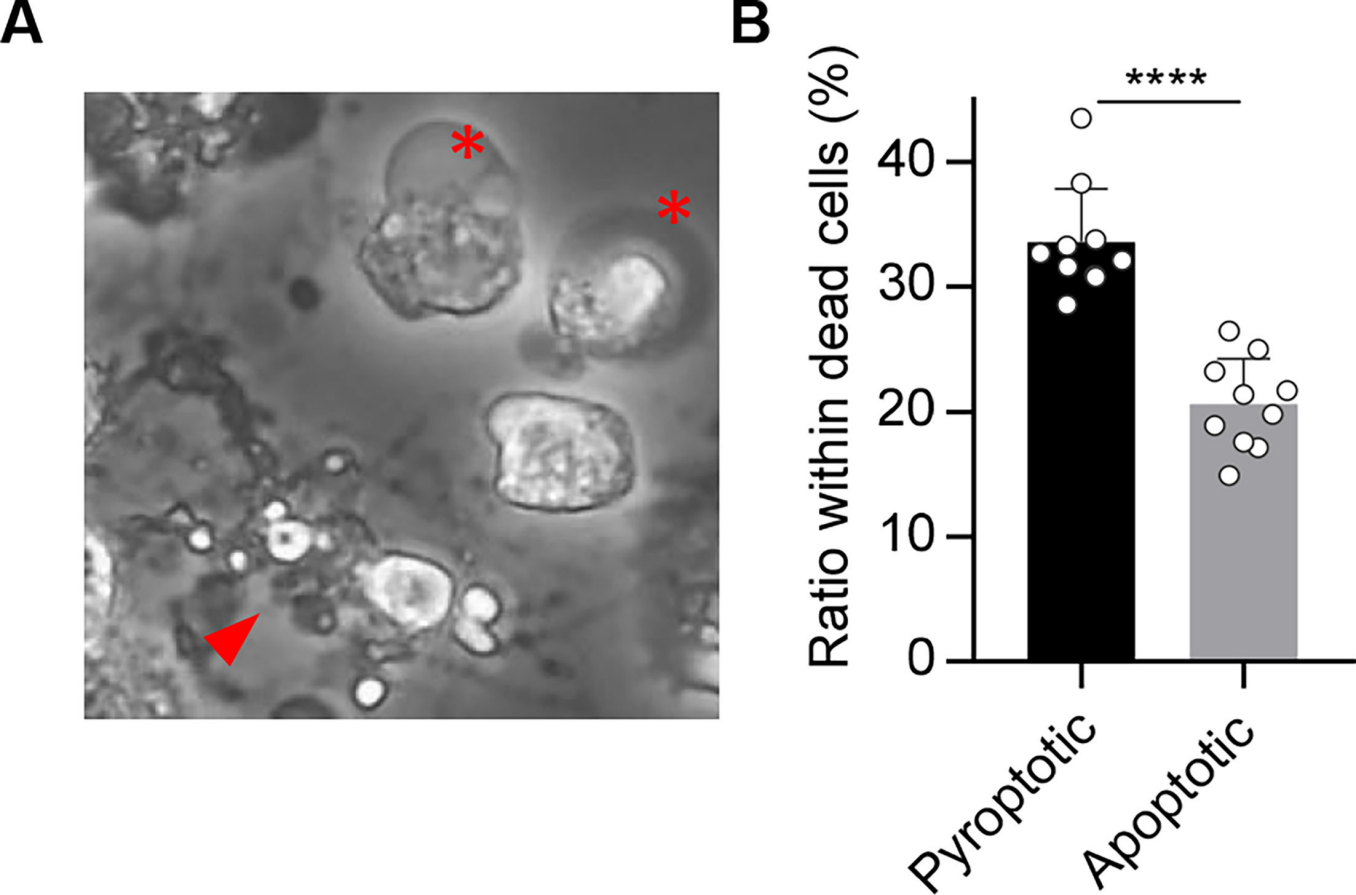
IAV infection induces distinct cell death morphologies in macrophages. (**A, B**) THP-1 macrophages were infected with IAV at an MOI of 10. At 24-h post-infection, dead cells were stained with PI and morphologically categorized (*n* > 100 cells; fields > 4). Asterisks and arrowheads indicate pyroptotic and apoptotic bodies, respectively. ****P < 0.0001; two-tailed Student’s *t*-test. Means ± SD from two independent experiments.

**Fig 2:**
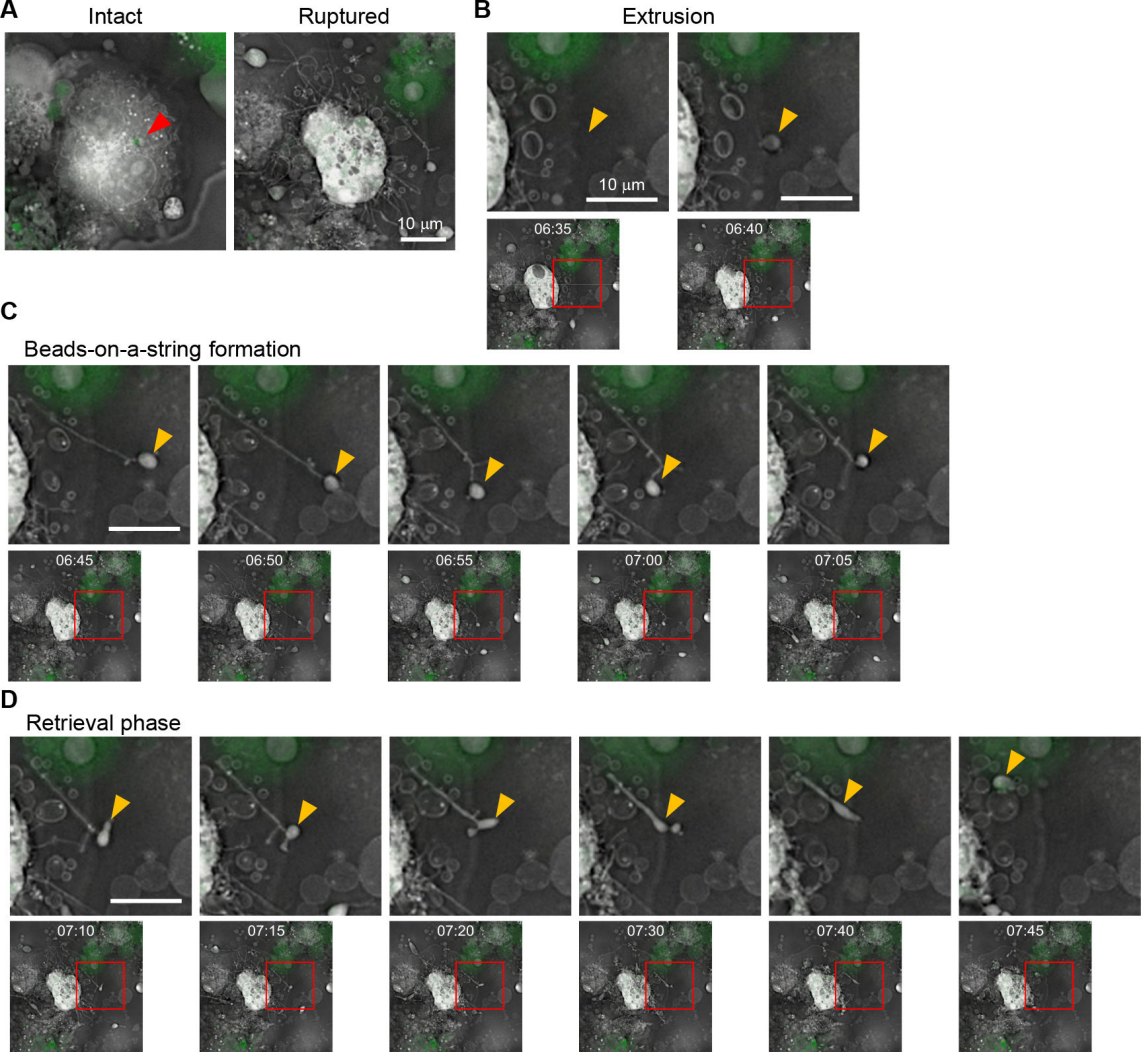
Time-lapse imaging of beaded apoptopodia formation using holotomographic microscope. THP-1 macrophages stably expressing GFP-ASC were infected with IAV at an MOI of 10. At 16-h post-infection, time-lapse imaging analysis was performed at 37°C with 5% CO_2_ using a holotomographic microscope at 5-min intervals (Movie S2). Representative time-lapse images show the intact and ruptured cells (**A**), extrusion of apoptotic bodies (**B**), extension of beaded apoptopodia (**C**), and retrieval of apoptotic bodies (**D**). The third image in panel **C** corresponds to the same “Ruptured” cell shown in panel **A**; panel **C** presents a magnified sequential series of still images. The red arrowhead indicates ASC speck formation. Yellow arrowheads indicate the same apoptotic body throughout the time course. Red boxes highlight the region of interest shown in the corresponding enlarged panels below. Time stamps indicate hours and minutes after the start of imaging. Scale bars, 10 µm.

To investigate how distinct cell death pathways manifest in IAV-infected macrophages, THP-1 macrophages were infected with A/Puerto Rico/8/1934 strain at a multiplicity of infection (MOI) of 10, and the dead cells were morphologically categorized to quantify the contribution of each pathway at 24-h post-infection (Fig. 1A and B). Approximately 40% of dead cells exhibited cellular swelling and plasma membrane ballooning, resembling “ghost” cells, a hallmark of pyroptosis, driven by osmotic lysis downstream of gasdermin pore formation (31). In contrast, about 19% of dead cells showed membrane protrusions that fragmented into apoptotic bodies, forming a “beads-on-a-string” membrane structure known as “beaded apoptopodia,” indicating apoptotic cell death (32). The remaining ~40% of dead cells were propidium iodide (PI)-positive but had not yet displayed overt plasma membrane rupture, representing a population in which the execution phase of cell death was not morphologically evident at the time of observation. These results confirm that pyroptosis is the predominant form of cell death in THP-1 macrophages upon IAV infection, while a smaller subset produces apoptotic bodies. Considering this divergence arises within a clonally derived cell population, we then investigated the regulation of apoptotic body formation in THP-1 macrophages using holotomographic microscopy, a technique that allows label-free visualization of cellular membranes and cytoskeletal structures by detecting refractive index differences (Fig. 2). THP-1 macrophages stably expressing GFP-ASC were infected with IAV at an MOI of 10, under which condition nearly all cells were infected (Fig. S1). THP-1 macrophages exhibiting ASC speck (Fig. 2A, arrowhead), a hallmark of inflammasome-mediated programmed cell death (33), were subjected to time-lapse imaging analysis at 16-h post-infection at 5-min intervals for 10 h. More than 90% of THP-1 macrophages exhibiting ASC speck formation were consistently positive for viral antigen (Fig. S2). Movie S1 shows that a representative macrophage underwent rapid cell swelling after forming ASC specks, followed by a marked expansion of the plasma membrane (Movie S1). This sequence of events corresponds to the “ghost cell” morphology described in Fig. 1. In contrast, some of the ASC-positive macrophages did not swell (Fig. 2A; Movie S2) and extruded membrane vesicles from the plasma membrane (Fig. 2B, arrowhead). These cells subsequently extended cytoplasmic filaments that elongated over tens of micrometers and connected to the vesicles, resulting in a characteristic beads-on-a-string appearance, termed beaded apoptopodia (Fig. 2C). Most vesicles partially pinched off as apoptotic bodies, but they remained transiently tethered and were frequently pulled back toward the parent cell, where they either fused or were re-internalized (Fig. 2D). These results demonstrate that even within a clonally derived cell population, individual macrophages execute fundamentally different cell death pathways.

### Beaded apoptopodia consist of stable detyrosinated microtubules

**Fig 3:**
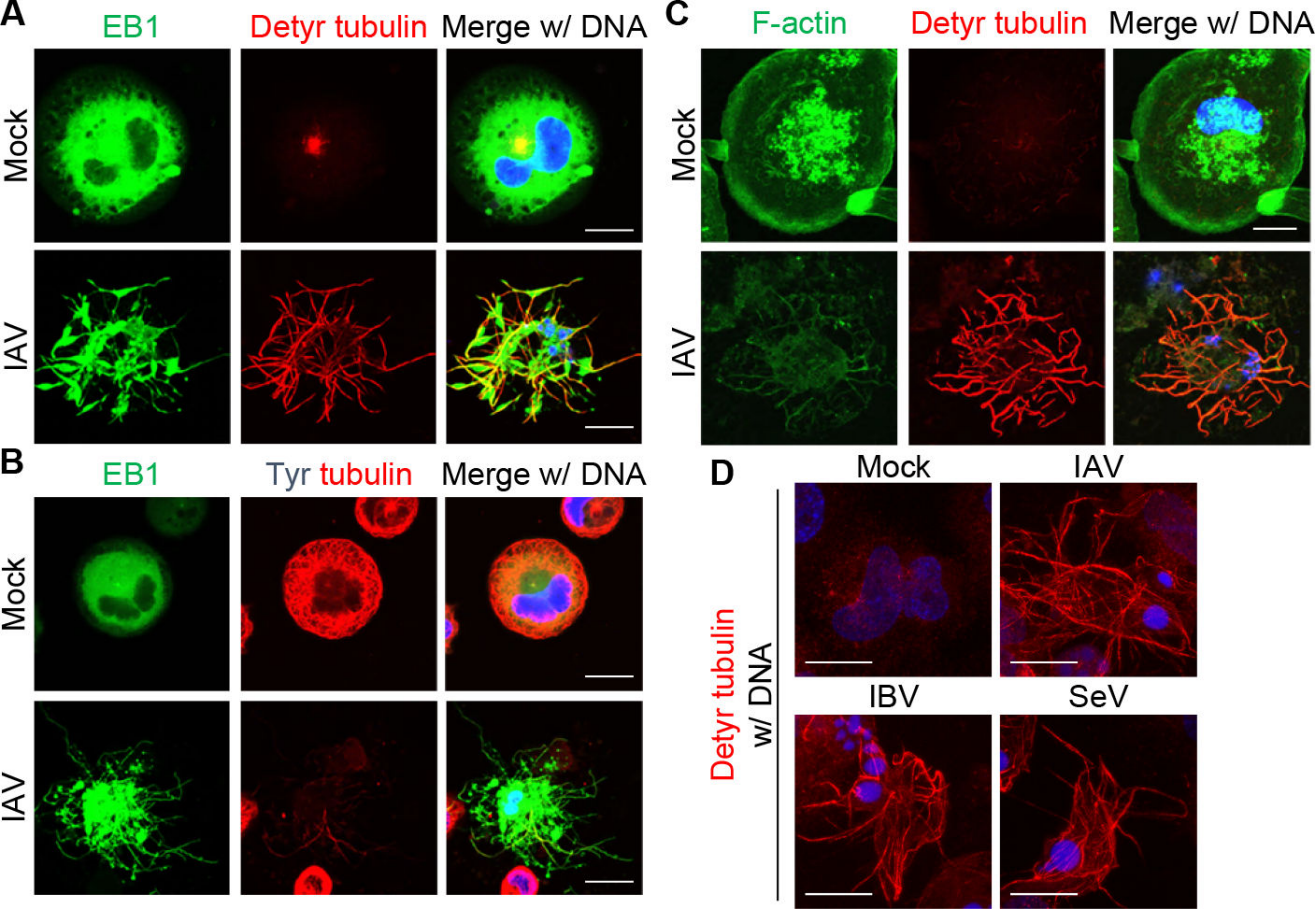
Beaded apoptopodia are composed of detyrosinated α-tubulin. (**A, B**) At 24-h post-infection, IAV-infected THP-1 macrophages stably expressing EB1-GFP were subjected to indirect immunofluorescence assays with antibodies against either detyr-tubulin (A, red) or tyr-tubulin (B, red). (**C**) At 24-h post-infection, IAV-infected THP-1 macrophages were immunostained with anti-detyrosinated tubulin antibody (red) and Alexa Fluor 488 phalloidin (green). (**D**) At 24-h post-infection, THP-1 macrophages infected with IAV, IBV, or SeV were immunostained with anti-detyrosinated tubulin antibody (red). Scale bars, 20 µm. DNA was counterstained with DAPI (blue). Data are representative of three independent experiments.

Although the beaded apoptopodia contain both microtubules and actin filaments, disrupting their dynamics with specific inhibitors—such as nocodazole or demecolcine for microtubules, and cytochalasin D or latrunculin A for actin filaments—had only limited effects on apoptopodia formation (34). To confirm the role of microtubules and actin filaments, we next performed indirect immunofluorescence assays using THP-1 macrophages stably expressing EB1-GFP, which is a microtubule plus-end marker (35), at 24-h post-infection (Fig. 3). In mock-treated macrophages, EB1-GFP was diffusely distributed in the cytoplasm, suggesting reduced microtubule dynamics (Fig. 3A). Following IAV infection, however, EB1-labeled microtubules reorganized into radial bundles that extended into the beaded apoptopodia (Fig. 3A) but not into pyroptotic cells (Fig. S3), confirming that the beaded apoptopodia contain microtubules as previously reported (Fig. 3A) (34, 36). Notably, these microtubules were co-stained with an antibody against detyr-tubulin (Fig. 3A), but not with one against tyrosinated α-tubulin (tyr-tubulin) (Fig. 3B). Detyr-tubulin marks long-lived, mechanically stable polymers, and tyr-tubulin marks dynamic ones (17, 37, 38). In mock-treated macrophages, the microtubule network was predominantly composed of tyr-tubulin, whereas the detyr-tubulin was present only in a minor subset of the total microtubule population (Fig. 3A and B). In contrast, the proportion of detyr-tubulin increased dramatically and was largely devoid of tyr-tubulin in infected macrophages. All cells exhibiting microtubule protrusions were positive for viral antigen, indicating that this response occurs specifically in infected cells (Fig. S4). These findings indicate that IAV infection promotes the detyrosination of α-tubulin, leading to the formation of stable microtubules within the beaded apoptopodia. In addition, we observed partial co-localization of F-actin with detyr-tubulin, suggesting that actin dynamics may play a limited role in the formation of beaded apoptopodia (Fig. 3C). To examine whether the detyrosination of α-tubulin also occurs in viral infections other than IAV, we analyzed cells infected with influenza B virus (IBV: B/Shanghai/361/2002) and Sendai virus (SeV: strain Z). In both cases, we observed increased detyrosination of α-tubulin and the formation of protruding structures in infected THP-1 macrophages (Fig. 3D). These results suggest that the virus-induced tubulin detyrosination and the associated morphological changes are not unique to IAV but are also induced by infection with other viruses.

**Fig 4:**
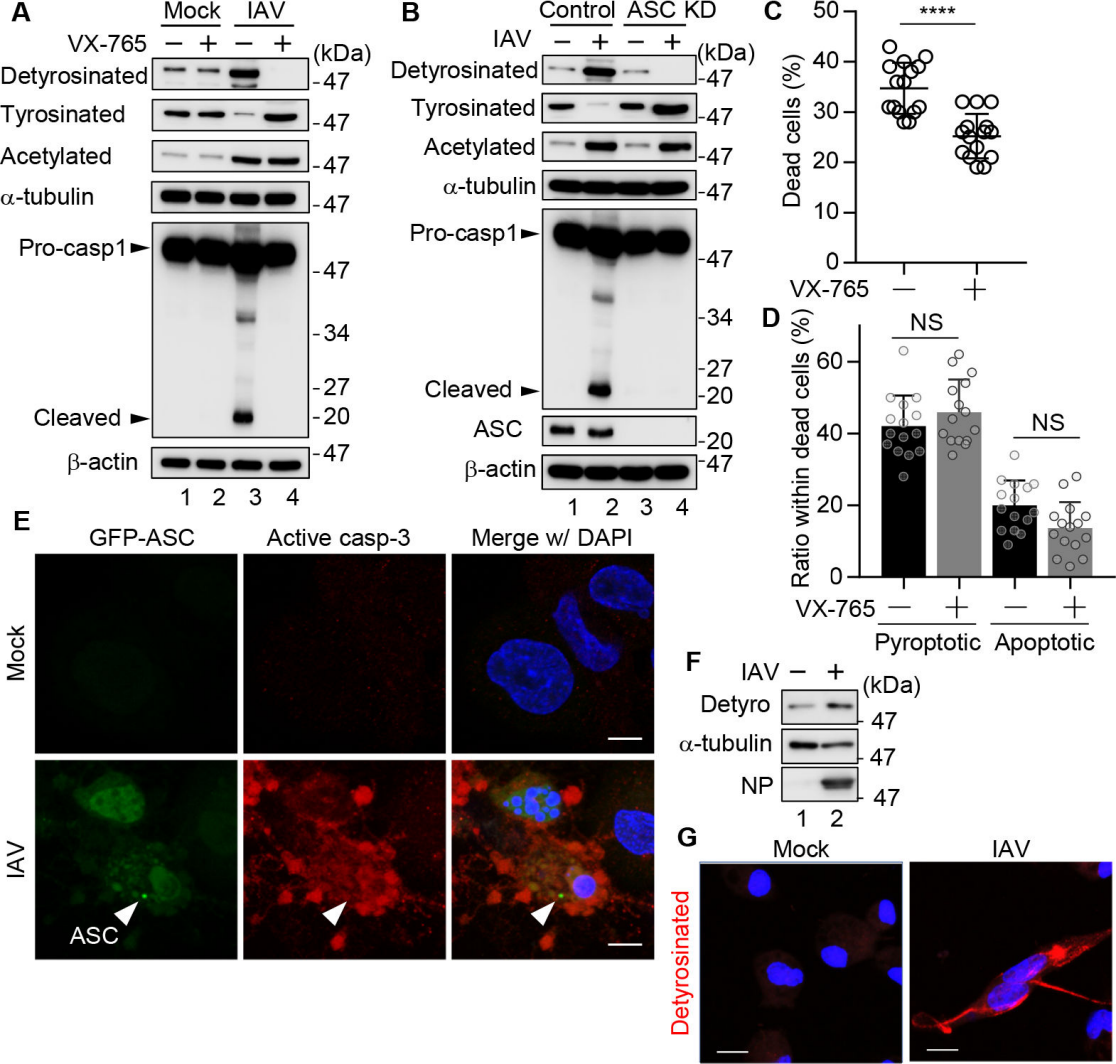
IAV infection induces detyrosination of α-tubulin in an inflammasome-dependent manner. (**A**) IAV-infected THP-1 macrophages were treated with or without 10 µM VX-765. At 24-h post-infection, cell lysates were subjected to western blot assays with the indicated antibodies. (**B**) THP-1 macrophages were transfected with scrambled siRNA (control) or ASC siRNA (ASC KD). At 48-h post-transfection, cells were infected with IAV at an MOI of 10. At 24-h post-infection, cell lysates were analyzed by western blot assays with the indicated antibodies. (**C and D**) THP-1 macrophages were infected with IAV at an MOI of 10 in the presence of 10 µM VX-765. At 24-h post-infection, dead cells were stained with PI, and dead cells, defined as either PI-positive or morphologically apoptotic/pyroptotic despite being PI-negative, were quantified (**C**). The dead cells were further categorized morphologically as described in Fig. 1 (*n* > 100 cells; fields > 15) (**D**). ****P < 0.0001; two-tailed Student’s *t*-test. Means ± SD from two independent experiments. (**E**) At 24-h post-infection, IAV-infected THP-1-GFP-ASC macrophages were immunostained with anti-caspase-3 antibody (red). Arrowhead indicates ASC specks. (**F and G**). At 24-h post-infection, IAV-infected primary peritoneal macrophages were subjected to western blot assays (**F**) and indirect immunofluorescence assays (**G**) with indicated antibodies. Data are representative of three independent experiments. Scale bars, 10 µm. DNA was counterstained with DAPI (blue).

Although the formation of ASC specks, an indicator of inflammasome activation, is generally associated with caspase-1-mediated pyroptotic cell death, a subset of ASC speck-positive cells exhibited beaded apoptopodia (Fig. 2). We next examined whether the detyrosination of microtubules depends on caspase-1 and ASC (Fig. 4). At 24-h post-infection, IAV infection promoted the detyrosination of α-tubulin, accompanied by a reciprocal reduction of tyr-tubulin. Acetylation of α-tubulin, another PTM associated with stabilized microtubules (18), was also enhanced by IAV infection. These findings suggest that IAV infection promotes microtubule stabilization through multiple mechanisms. However, VX-765 treatment, a potent inhibitor of caspase-1 (39), specifically suppressed the detyrosination of α-tubulin without affecting its acetylation level (Fig. 4A). Similarly, IAV-induced detyrosination of microtubules was also suppressed in ASC knockdown cells (Fig. 4B). These results indicate that the microtubule detyrosination upon IAV infection occurs downstream of inflammasome activation. We next morphologically categorized dead cells in the presence of VX-765 at 24-h post-infection. VX-765 treatment reduced the total number of dead cells (Fig. 4C), but did not significantly alter the proportions of pyroptotic and apoptotic cell death (Fig. 4D). When we stained for activated caspase-3, a canonical marker of apoptosis, some ASC speck–positive cells with beaded apoptopodia were also positive for activated caspase-3 (Fig. 4E). These observations revealed that the inhibition of pyroptosis concomitantly suppresses apoptosis, suggesting that these two cell death pathways are mechanistically interconnected in IAV-infected macrophages. Notably, a similar detyrosination response was observed in primary peritoneal macrophages isolated from mice, confirming that this PTM is not restricted to a particular cell line (Fig. 4F and G). Although the detyrosination signal appeared weaker in primary macrophages, this is likely due to the lower infection efficiency in primary macrophages compared with THP-1 macrophages (Fig. S1).

### VASH1 is responsible for the microtubule detyrosination upon IAV infection

**Fig 5:**
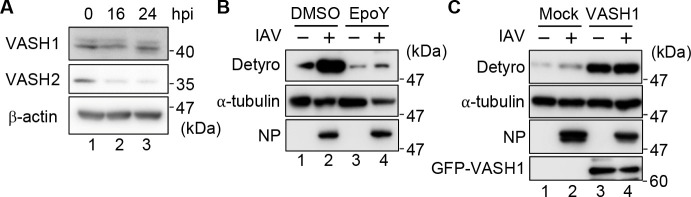
VASH1 mediates detyrosination of α-tubulin upon IAV infection. (**A**) At 0-, 16-, and 24-h post-infection, cell lysates obtained from THP-1 macrophages were analyzed by western blot with the indicated antibodies. (**B**) IAV-infected THP-1 macrophages were treated with or without 20 µM EpoY. At 24-h post-infection, cell lysates were analyzed by western blot assays with the indicated antibodies. (**C**) THP-1 macrophages overexpressing GFP-VASH1 were infected with IAV at a MOI of 10. At 24-h post-infection, cell lysates were analyzed by western blot assays with the indicated antibodies. Data are representative of three independent experiments.

Tubulin carboxypeptidases known as VASH1 and VASH2 have been identified as the enzymes responsible for microtubule detyrosination (23, 25). Since IAV infection induces microtubule detyrosination, we next examined whether VASH1 and VASH2 are involved in this process. Western blot revealed that the expression level of VASH1 remained steady at both 16-h and 24-h post-infection, whereas VASH2 expression declined upon IAV infection (Fig. 5A), suggesting that VASH1 is the dominant detyrosination enzyme during IAV-mediated cell death. To further confirm the involvement of VASH1 in the microtubule detyrosination upon IAV infection, infected THP-1 macrophages were treated with 20 µM EpoY, a selective inhibitor of VASH1 (23). As expected, EpoY treatment inhibited IAV-mediated microtubule detyrosination (Fig. 5B). Importantly, viral nucleoprotein (NP) expression remained unaffected, indicating that EpoY did not impair the infection efficiency of IAV. Conversely, overexpression of VASH1 (GFP-VASH1) further increased the IAV-mediated microtubule detyrosination (Fig. 5C). These results suggest that VASH1 serves as the major tubulin carboxypeptidase responsible for microtubule detyrosination in IAV-infected macrophages.

**Fig 6:**
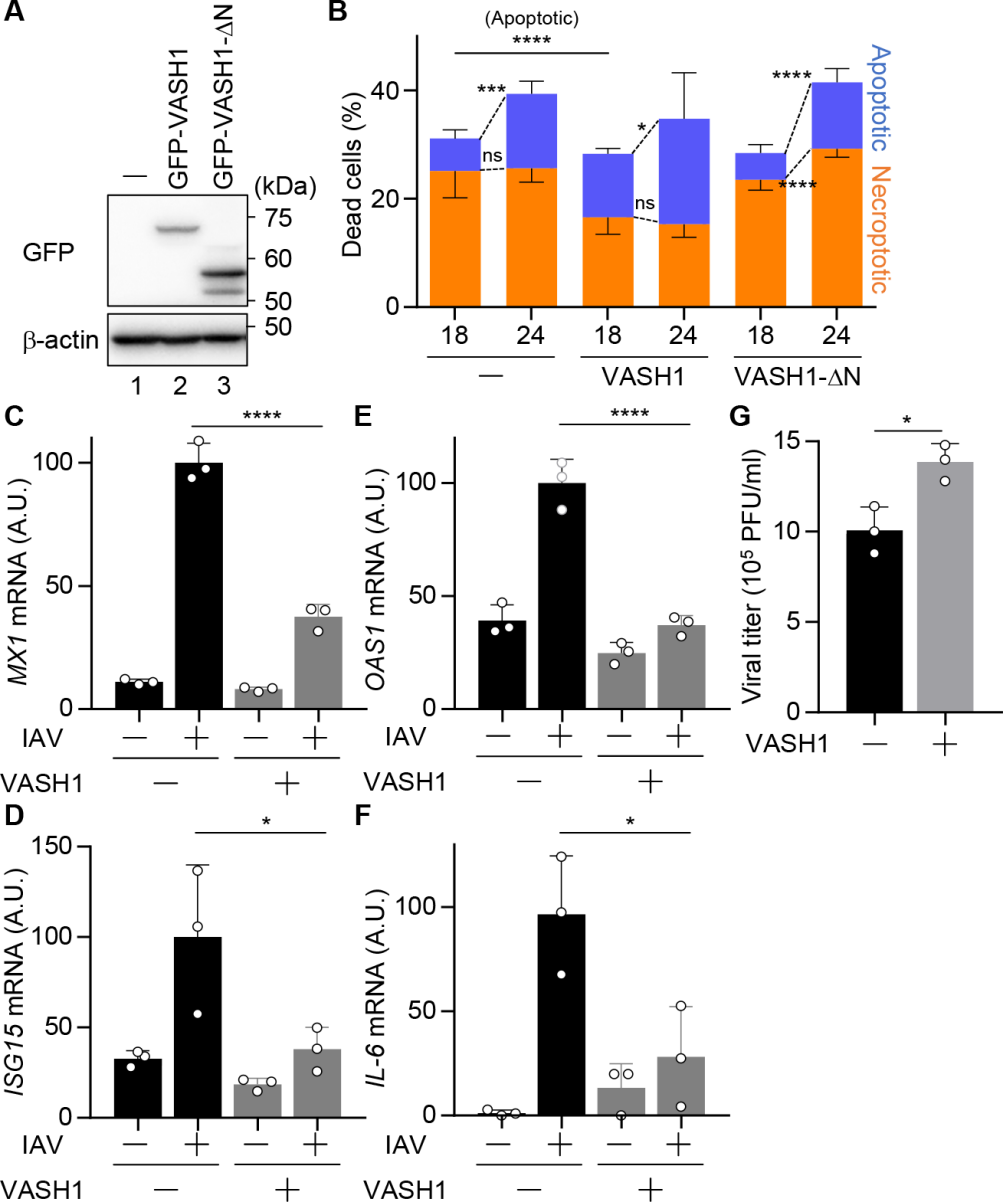
Increased α-tubulin detyrosination induces the formation of beaded apoptopodia. Wild-type THP-1 macrophages and those overexpressing GFP-VASH1 or GFP-VASH1ΔN were infected with IAV at an MOI of 10. (**A**) The cell lysates were analyzed by western blot assays with anti-GFP and anti-β-actin antibodies. Data are representative of two independent experiments. (**B**) At 18- and 24-h post-infection, dead cells were stained with PI, and dead cells, defined as either PI-positive or morphologically apoptotic (blue)/pyroptotic (orange) despite being PI-negative, were quantified (*n* > 100 cells; fields > 3). (**C–F**) At 24-h post-infection, total RNAs were purified and subjected to reverse transcription followed by real-time PCR with primers specific for *MX1* (**C**), *ISG15* (**D**)*, OAS1* (**E**), and *IL-6* genes (**F**), respectively. (**G**) At 24-h post-infection, the viral titers were determined by plaque assays with MDCK cells. *P < 0.05, **P < 0.01, ****P < 0.0001; two-tailed Student’s *t*-test. Means ± SD from three independent experiments (**B–G**).

To test whether the IAV-induced detyrosinated microtubules influence the mode of cell death, we analyzed THP-1 macrophages constitutively overexpressing GFP-VASH1 or a VASH1 mutant (GFP-VASH1ΔN) lacking the N-terminal region (residues 1–112) required for its enzymatic activity (Fig. 6A). At 18- and 24-h post-infection, the number and morphology of dead cells were quantified. VASH1 overexpression did not change the total number of dead cells, as determined by PI staining (Fig. 6B). In the absence of VASH1 overexpression, pyroptosis was the predominant form of cell death, but apoptotic cell death increased with the progression of infection (Fig. 6B; orange, necroptotic; blue, apoptotic). In contrast, VASH1-expressing cells exhibited reduced pyroptotic death, and apoptotic cell death became predominant (Fig. 6B). In VASH1ΔN-expressing macrophages, the proportions of pyroptotic and apoptotic cells remained unchanged, indicating that the observed effects depended on VASH1 enzymatic activity rather than non-specific overexpression. Since apoptotic cell death is an immunologically silent cell death pathway, we also examined antiviral and inflammatory gene expression, such as *MX1*, *ISG15*, *OAS1*, and *IL-6* (Fig. 6C through F), as well as viral titers (Fig. 6G). The expression levels of these genes were reduced to approximately 30% of wild-type levels in VASH1-overexpressing cells (Fig. 6C through F), possibly due to a shift from pyroptotic to apoptotic cell death. Consistent with these findings, viral titers were modestly but reproducibly increased in VASH1-overexpressing cells compared with wild-type cells (Fig. 6G).

## DISCUSSION

We found that microtubule detyrosination by VASH1 occurs in a caspase-1-dependent manner (Fig. 4) and is activated concurrently with the onset of cell death (Movie S2). VASH1 catalyzes the removal of the C-terminal tyrosine of α-tubulin and requires dimerization with SVBP to become enzymatically active (24, 40). While the precise mechanism by which caspase-1 signaling activates the VASH1-SVBP complex remains unclear, proteolytic cleavage or PTM downstream of inflammasome activation may regulate VASH1 activity. Notably, the carboxypeptidase activity of VASH1 is known to be activated in response to various stress signals, such as VEGF and bFGF, and functions as a negative regulator of angiogenesis in endothelial cells, suggesting that it serves as a stress- or stimulation-responsive effector (41, 42). In contrast, VASH2 expression was significantly downregulated upon IAV infection in our system (Fig. 5A), suggesting that it is dispensable under these conditions. Despite its similarity to VASH1, VASH2 exhibits distinct expression patterns and functional characteristics. VASH2 is predominantly expressed in mononuclear cells rather than endothelial cells and generally promotes angiogenesis (43), in contrast to the anti-angiogenic role of VASH1 (44). Furthermore, VASH2 has lower microtubule diffusibility and is thought to mediate more localized detyrosination, whereas VASH1 induces broader, global microtubule detyrosination (45).

Programmed cell death, such as apoptosis, involves extensive cytoskeletal remodeling to ensure proper morphological transformation and clearance of dying cells (46). Executioner caspases, such as caspase-3, caspase-6, and caspase-7, play pivotal roles in this process by cleaving key cytoskeletal components (47). For instance, caspase-3 cleaves actin-binding proteins, such as gelsolin and spectrin, to promote actin network disassembly and membrane blebbing (48, 49). Caspase-6 also cleaves α-tubulin, destabilizing microtubules and contributing to neurodegenerative disease (50). Caspase-mediated cleavage of intermediate filaments, including vimentin, keratins, and lamins, also contributes to cytoskeletal collapse (51–53). In contrast, caspase-1 is primarily known for its role in pyroptosis through gasdermin D (GSDMD) cleavage (54), and its direct role in cytoskeletal remodeling is not well understood. Our findings demonstrated the involvement of caspase-1 in regulating microtubule modifications, particularly detyrosination via VASH1. This highlights a new mechanistic link between inflammasome signaling and microtubule remodeling.

**Fig 7:**
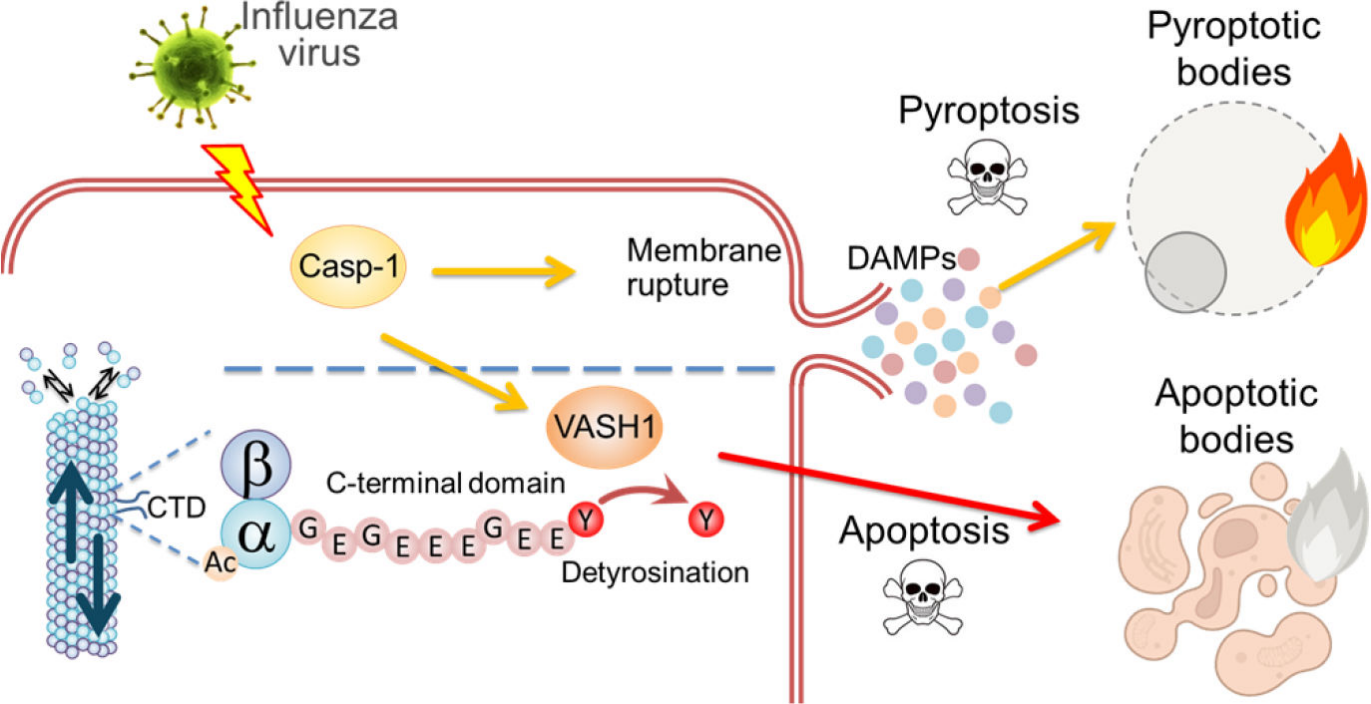
Proposed model. Influenza virus infection causes pyroptotic and apoptotic cell death in a caspase-1-dependent manner. While caspase-1 activation is typically associated with pyroptosis via membrane pore formation, caspase-1 also triggers apoptotic pathways in parallel or as a compensatory mechanism. This switch from pyroptosis to apoptosis requires the detyrosination of α-tubulin by VASH1 to stabilize microtubules and promote apoptotic morphology.

We found that even in IAV-infected macrophages with ASC speck formation, a hallmark of inflammasome activation, a subset of cells underwent apoptotic cell death (Fig. 7). While inflammasome activation is typically associated with pyroptosis via GSDMD pore formation, our results suggest that caspase-1 may also trigger apoptotic pathways in parallel or as a compensatory mechanism. Consistent with this, previous studies have demonstrated that in the absence of GSDMD, inflammasome activation induces apoptosis in macrophages through caspase-3 activation. This supports a model in which caspase-1, under certain conditions, initiates apoptotic signaling via intermediates such as BID, caspase-9, or caspase-6, providing an alternate route of cell death (55–57). Notably, cells with low GSDMD expression (e.g., neurons, mast cells) often undergo apoptosis rather than pyroptosis in response to inflammasome stimuli (57). However, it is reported that GSDMD is directed to the plasma membrane through palmitoylation at cysteine residues, possibly in a microtubule-independent manner (58, 59). Therefore, it is unlikely that detyr-tubulin directly mediates the intracellular transport of GSDMD to the plasma membrane. This switch from pyroptosis to apoptosis may represent a host-protective feedback mechanism to limit excess inflammation because apoptosis preserves membrane integrity and facilitates immunologically silent clearance by phagocytic cells (7, 36, 60). Our findings suggest that the VASH1-SVBP axis contributes to such regulation by stabilizing microtubules and promoting apoptotic morphology. Targeting VASH1-mediated microtubule detyrosination may offer a novel strategy to modulate cell death outcomes during infection and inflammation.

## MATERIALS AND METHODS

### Biological materials

IAV A/Puerto Rico/8/34, IBV B/Shanghai/361/2002, and SeV strain Z were grown at 35.5°C for 48 h in allantoic sacs of 11-day-old embryonated eggs and stored at −80°C until use (61). Mouse monoclonal antibodies against GFP (Nacalai Tesque; 04363-66), tyrosinated tubulin (SIGMA; TUB-1A2), acetylated tubulin (SIGMA; 6-11B-1), α-tubulin (SIGMA; DM1A), β-actin (SIGMA; A5441), ASC (Merck Millipore; 04-147), caspase-1 (R&D systems; MAB6215), and VASH2 (Proteintech; 67753-1-Ig), and rabbit polyclonal antibodies against detyrosinated tubulin (Merck Millipore; AB3201) and VASH1 (Proteintech; 12730-1-AP) were purchased. A rabbit polyclonal antibody against NP was prepared as previously described (61). EpoY (SIGMA; SML2301-5MG) and VX-765 (Chemietek; CT-VX765) were purchased. For the construction of a plasmid expressing VASH1, total RNA was reverse-transcribed as a template using the oligo(dT)_20_ primer, and the cDNA was amplified with the primers 5′-GACGAGCTGTACAAGATGCCAGGGGGGAAGAAAGGTGGCTGGGGGT-3′ and 5′-GATCCTTGCGGCCGCTCAGACCCGGATCTGGTACCCGT-3′, and then cloned into the pCDH plasmid using Gibson Assembly reagent (New England Biolabs; E5510S). The cDNA of VASH1ΔN was amplified from pCDH-GFP-VASH1 as a template using the primers (5′-CCGTCTACACCTGTCCCTGA-3′ and 5′-GGGACAGGTGTAGACGGCTTGTACAGCTCGTCCATGC-3′) and circularized by Gibson Assembly reagent. The production of the lentivirus was carried out according to the manufacturer’s protocol. The lentivirus carrying EB1-GFP or GFP-ASC was prepared as previously described (61, 62).

### Cell culture

THP-1 cells were purchased from the RIKEN Bioresource Research Center and were grown in RPMI 1640 medium (Gibco; 11875119) containing 10% fetal bovine serum. THP-1 cells were transduced with the lentivirus carrying VASH1 (THP-1-VASH1 cells), EB1-GFP (THP-1-EB1 cells), or GFP-ASC (THP-1-ASC cells). THP-1 cells were differentiated into macrophages by incubating for 24 h with 100 ng of phorbol 12-myristate 13-acetate (Nacalai Tesque; 27547-14) with 20% fetal bovine serum. THP-1 macrophages were infected with A/Puerto Rico/8/34, B/Shanghai/361/2002, or SeV at a MOI of 10 for 1 h in RPMI medium without fetal bovine serum (FBS). After washing with RPMI medium, the THP-1 macrophages were further incubated in RPMI medium containing 10% FBS. Primary peritoneal macrophages were isolated from 8- to 10-week-old C57BL/6 mice at 72-h post-treatment of 2 mL sterile 2.5% Brewer thioglycollate (BD, 211716) into the peritoneal cavity. The siRNA against *Asc* gene was purchased (Thermo Fisher Scientific). Cells were transfected with siRNA using Lipofectamine RNAi Max (Thermo Fisher Scientific) according to the manufacturer’s protocol. As a negative control, non-specific scrambled siRNA was used.

### Quantification of cell death

Cell death was examined by a combination of morphological assessment and PI exclusion. Infected cells were incubated with 1 µg/mL PI for 10 min at room temperature, and PI-positive cells were counted using a fluorescence microscope. To distinguish the modes of cell death, apoptotic bodies and pyroptotic bodies were manually identified based on characteristic morphological features under phase-contrast microscopy (*N* > 100 cells/field). Cell death was examined by a combination of morphological assessment and PI exclusion. Infected cells were incubated with 1 µg/mL PI for 10 min at room temperature, and PI-positive cells were counted using a fluorescence microscope. To distinguish the modes of cell death, apoptotic bodies and pyroptotic bodies were manually identified based on characteristic morphological features under phase-contrast microscopy (*N* > 100 cells/field). Apoptotic bodies were defined by membrane blebbing and cellular fragmentation, whereas pyroptotic bodies were defined by cell swelling, followed by the formation of a ghost cell-like morphology. At least seven randomly selected fields per sample were analyzed from two independent experiments, and the results were expressed as percentages of total dead cells.

### Indirect immunofluorescence assays

THP-1-EB1 macrophages seeded on glass coverslips treated with 0.002% poly-L-lysine (SIGMA) were infected with IAV at a MOI of 10. At 24-h post-infection, cells were fixed with 4% paraformaldehyde for 10 min and permeabilized with 0.5% Triton X-100 in phosphate-buffered saline (PBS) for 5  min. After incubating with PBS containing 1% non-fat milk for 30  min, the coverslips were incubated with primary antibodies for 1 h, followed by Alexa Fluor 488- and 568-conjugated secondary antibodies, respectively (Thermo Fisher Scientific). Alexa Fluor 488-conjugated phalloidin was used to stain F-actin. Images were acquired using a confocal laser scanning microscope (LSM700; Carl Zeiss) using ×63 apochromat objective (NA = 1.4). All images are presented as maximum intensity projections.

### Label-free imaging analysis

THP-1-ASC macrophages were infected with IAV at an MOI of 10. At 16-h post-infection, time-lapse imaging analysis was performed at 37°C with 5% CO_2_ using a holotomographic microscope (Tomocube; HT-X1) at 5-min intervals for 10 h.

### Western blotting analysis

The cell lysates were separated using sodium dodecyl sulfate polyacrylamide gel electrophoresis and transferred onto a polyvinylidene difluoride membrane. The membrane was blocked with 5% non-fat milk for 30 min at room temperature and incubated with the primary antibodies at room temperature for 1 h. The membranes were subsequently incubated with HRP-conjugated secondary antibodies (Cell Signaling Technology) for 10 min. The signals were detected using the FUSION chemiluminescence and fluorescence imaging system (Vilber-Lourmat).

### RNA analysis

The RNA amounts of the *MX1*, *ISG15*, *OAS1*, and *IL-6* genes were examined by RT-qPCR. Purified total RNAs were reverse-transcribed with oligo(dT)_20_ primer and 18S rRNA primer (5′-GGGAGTGGGTAATTTGCGC-3′) and subjected to quantitative PCR using FastStart SYBR Green (Roche) with the primer sets for *MX1* (5ʹ-GGCTGTTTACCAGACTCCGACA-3ʹ and 5ʹ-CACAAAGCCTGGCAGCTCTCTA-3ʹ), *ISG15* (5ʹ-TCCTGGTGAGGAATAACAAGGG-3ʹ and 5ʹ-CTCAGCCAGAACAGGTCGTC-3ʹ), *OAS1* (5ʹ-GAAGCTGCCACCTCAGTAT-3ʹ and 5ʹ-GCTGCCTTCTCAGGTACTTT-3ʹ), *IL-6* (5ʹ-CAAATTCGGTACATCCTCGACGGC-3ʹ and 5ʹ-GGTTCAGGTTGTTTTCTGCCAGTGC-3ʹ), and 18S rRNA (5ʹ-AACGGCTACCACATCCAAGG-3ʹ and 5ʹ-GGGAGTGGGTAATTTGCGC-3ʹ). The results were normalized to the level of 18S rRNA.

### Statistical analysis

The statistical significance of experimental results was determined using unpaired two-tailed Student’s *t*-test, one-way analysis of variance with Tukey’s test using GraphPad Prism (version 7.03). ns, not significant. **** *P* < 0.0001, **P < 0.01, *P  <  0.05.

## Data Availability

All data supporting the findings of this study are available within the main text and supplemental material.

## SUPPLEMENTAL MATERIAL

Supplemental text jvi.01847-25-s0001.docx Legends for Movies S1 and S2; Fig. S1 to S4.

Movie S1 jvi.01847-25-s0002.avi Time-lapse images illustrating pyroptotic bodies in THP-1 macrophages stably expressing GFP-ASC and infected with IAV.

Movie S2 jvi.01847-25-s0003.avi Time-lapse images illustrating beaded apoptopodia in THP-1 macrophages stably expressing GFP-ASC and infected with IAV.

ASM does not own the copyrights to Supplemental Material that may be linked to, or accessed through, an article. The authors have granted ASM a non-exclusive, world-wide license to publish the Supplemental Material files. Please contact the corresponding author directly for reuse.
