# Supplementary material for: Microtubule detyrosination links inflammasome activation to apoptotic cell death in macrophages upon influenza A virus infection
Source: J Virol. 2025 Dec 9;100(1):e01847-25. doi: 10.1128/jvi.01847-25 (PMC12817897; doi:10.1128/jvi.01847-25)
Supplement: Supplemental text — Legends for Movies S1 and S2; Fig. S1 to S4. [file jvi.01847-25-s0001.docx]

**Title:**

Microtubule detyrosination links inflammasome activation to apoptotic cell death in macrophages upon influenza A virus infection

**Authors:**

Joyeeta Kar^1, 8^, Mikako Hirohama^2, 8^, Kotono Nakayama^3^, SangJoon Lee^2, 4^, Atsushi Kawaguchi^1, 2, 3, 5, 6, 7, 8^*

^1^ Graduate School of Comprehensive Human Sciences, University of Tsukuba, Tsukuba 305-8575 Japan

^2^ Department of Infection Biology, Institute of Medicine, University of Tsukuba, Tsukuba 305-8575 Japan

^3^ School of Medicine, University of Tsukuba, Tsukuba 305-8575 Japan

^4^ Department of Biological Sciences, Ulsan National Institute of Science and Technology (UNIST), Ulsan 44919 Republic of Korea

^5^ Transborder Medical Research Center, University of Tsukuba, Tsukuba 305-8575 Japan

^6^ Microbiology Research Center for Sustainability, University of Tsukuba, Tsukuba 305-8575 Japan

^7^ Center for Quantum and Information Life Sciences, University of Tsukuba, Tsukuba 305-8575 Japan

^8^ These authors contributed equally to this work. The author order was determined in order of increasing seniority.

**Supplementary Movie 1**

THP-1 macrophages stably expressing GFP-ASC were infected with IAV at an MOI of 10. At 16 h post-infection, time-lapse imaging analysis was performed at 37°C with 5% CO_2_ using a holotomographic microscope at 5-min intervals. Representative time-lapse images illustrating pyroptotic bodies were shown.

**Supplementary Movie 2**

THP-1 macrophages stably expressing GFP-ASC were infected with IAV at an MOI of 10. At 16 h post-infection, time-lapse imaging analysis was performed at 37°C with 5% CO_2_ using a holotomographic microscope at 5-min intervals. Representative time-lapse images illustrating beaded apoptopodia were shown.

**
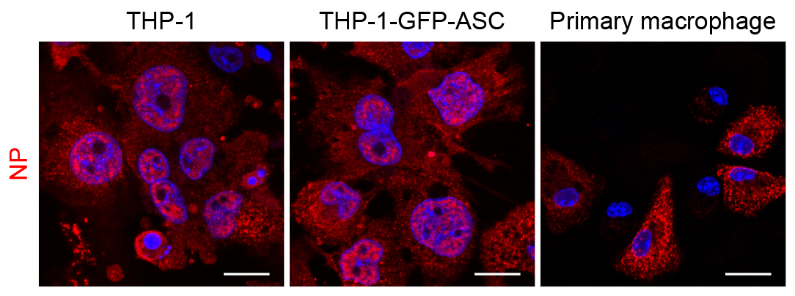
**

**Supplementary Figure 1**

THP-1 macrophages, THP-1-GFP-VASH1 macrophages, and primary peritoneal macrophages were infected with IAV at an MOI of 10. At 24 h post-infection, the cells were subjected to indirect immunofluorescence assays with anti-NP antibody (red). Data are representative of three independent experiments. Scale bars, 20 μm. DNA was counterstained with DAPI (blue).

**
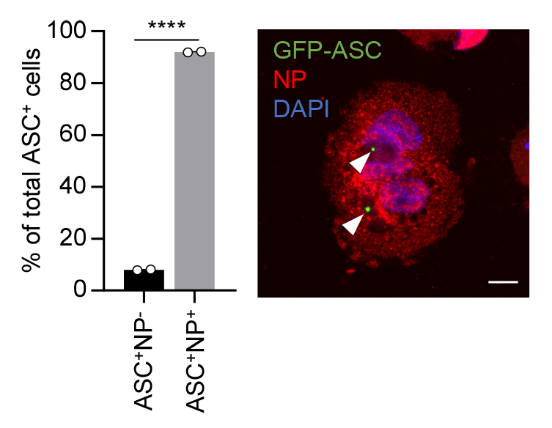
**

**Supplementary Figure 2**

THP-1-GFP-ASC macrophages were infected with IAV at an MOI of 10. At 24 h post-infection, the cells were subjected to indirect immunofluorescence assays with anti-NP antibody (red). Arrowheads indicate ASC speck formation (green). The percentages of ASC speck-positive cells that were unstained (black bar) or stained with NP (gray bar) are shown relative to the total number of ASC speck-positive cells (n > 80 cells). Data are representative of two independent experiments. Scale bars, 10 μm. DNA was counterstained with DAPI (blue).

**
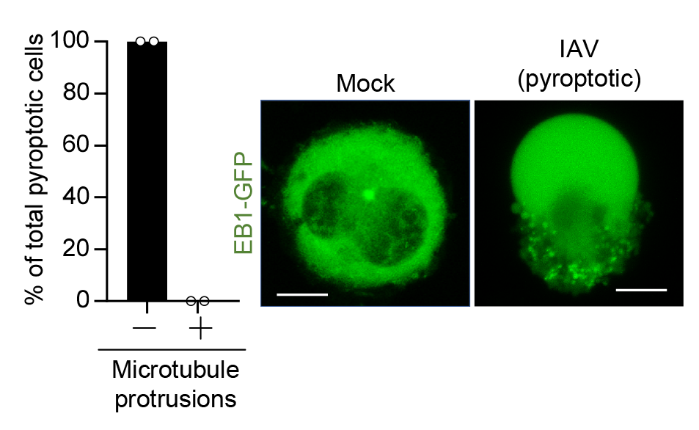
**

**Supplementary Figure 3**

THP-1-EB1-GFP macrophages were infected with IAV at an MOI of 10. At 24 h post-infection, images were acquired using a confocal laser scanning microscope. The percentages of pyroptotic cells with or without microtubule protrusions are shown (n > 30 cells). Data are representative of two independent experiments. Scale bars, 10 μm.

**
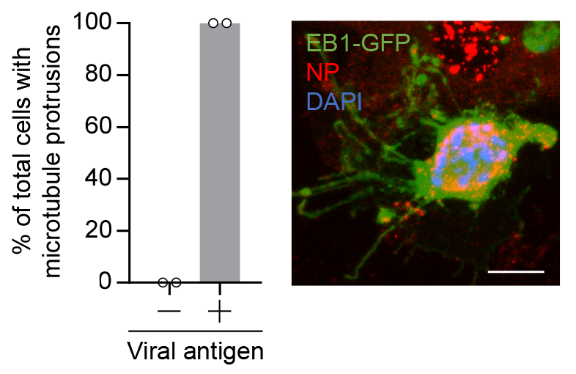
**

**Supplementary Figure 4**

THP-1-EB1-GFP macrophages were infected with IAV at an MOI of 10. At 24 h post-infection, the cells were subjected to indirect immunofluorescence assays with anti-NP antibody (red). The percentages of cells with or without NP signal are shown (n > 30 cells). Data are representative of two independent experiments. Scale bar, 10 μm.
